# Serum α-Klotho and SIRT1 - Relationship with graft function, inflammation and hospitalization rates in kidney transplant recipients

**DOI:** 10.3389/ti.2026.16186

**Published:** 2026-06-02

**Authors:** Anna Sączek, Krzysztof Batko, Małgorzata Banaszkiewicz, Jolanta Małyszko, Ewa Koc-Żórawska, Marcin Żórawski, Jacek Małyszko, Karolina Niezabitowska, Katarzyna Sobczyńska, Przemysław Miarka, Alina Bętkowska-Prokop, Katarzyna Krzanowska, Marcin Krzanowski

**Affiliations:** Department of Nephrology and Transplantology, Jagiellonian University Medical College, Kraków, Poland

**Keywords:** biomarker, hospitalization, inflammation, kidney, Klotho

## Abstract

Emerging evidence indicates kidney transplant recipients (KTRs) suffer from accelerated cellular aging, low-grade inflammation and variable degrees of allograft dysfunction. These pathobiological processes shape chronic kidney disease in the graft, for which α-Klotho is being explored as a candidate peptide marker for risk stratification. In this observational cohort study, we recruited 127 KTRs in outpatient care at the University Hospital in Kraków, Poland between September 2016 and June 2019. Serum α-Klotho, sirtuin-1 (SIRT1), and high-sensitivity interleukin-6 (hsIL-6) were assayed using ELISA kits in KTRs and 32 healthy controls (HCs). Thereafter, we utilized crude, age-adjusted, and fully adjusted Firth Poisson regression models with robust standard errors to evaluate predictors of all-cause hospitalization. We observed that circulating α-Klotho was reduced in KTRs, as compared with HCs (median 616 vs. 1,042 pg/mL, P < 0.001). Further, α-Klotho was moderately associated with eGFR at baseline (rho = 0.30) and final follow-up (rho = 0.29). In contrast, α-Klotho was inversely correlated with hsIL-6 (rho = −0.39, 95% CI −0.60–0.15, P = 0.002). In multivariable linear regression models, ln (α-Klotho) changes were tied to higher final eGFR (14.8 95% CI 6.2–25.6 mL/min/1.73 m^2^). During follow-up of 274 person-years, we recorded 153 hospitalizations. In multivariable models, higher ln (α-Klotho) was independently associated with lower hospitalization rate (IRR 0.31, 95% CI 0.15–0.65, P = 0.002). This association persisted after adjustment for baseline eGFR (IRR 0.37, 95% CI 0.20–0.69, P = 0.002). Overall, given further validation and standardization of assay technology, serum α-Klotho may be a strong candidate for supplemental, outpatient risk stratification in KTRs.

## Graphical Abstract

**Figure:**
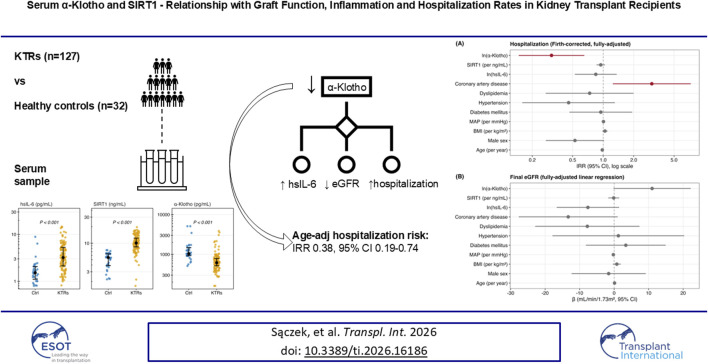

## Introduction

Patients with chronic kidney disease (CKD) and kidney transplant recipients (KTRs) experience enhanced biological aging, variable renal dysfunction and low-grade inflammation, which are drivers of cardiovascular (CV), infectious and neoplastic morbidity [1, 2]. Candidate prognostic markers, such as α-Klotho and sirtuin-1 (SIRT1), are increasingly studied as signature molecules of cytoprotective, anti-inflammatory and renoprotective pathways [2, 3]. The need for improved post kidney transplant (KTx) management, both from a perspective of graft dysfunction and comorbid disorder risk, has prompted research into risk stratification tools [4–7]. The post-KTx setting is unique in that chronic immunosuppression carries the risk of cardiometabolic side-effects that must be balanced against the risk of allograft dysfunction [8].

α-Klotho has been identified both as a transmembrane co-receptor for fibroblast growth factor-23 (FGF-23) and as a soluble circulating form [9, 10]. FGF-23 is known to regulate phosphate and vitamin D metabolism [11, 12], though Klotho also exerts anti-inflammatory and anti-fibrotic effects [13]. The kidney is considered a major source of α-Klotho [14–16], which strengthens its potential utility as a circulating marker. In murine models, lower α-Klotho is tied to premature aging, vascular disease, skin atrophy, and mineral-bone dysregulation [14]. In turn, its overexpression is tied to enhanced longevity, which may be mediated through effects on insulin and insulin-like growth factor-1 signalling pathways [17].

In humans, lower α-Klotho is tied to CV disease and mortality [18]. Population studies report inverse associations with body-mass measures and non-linear relationships with lipid abnormalities [18, 19]. Lower α-Klotho is also linked to metabolic syndrome, with apparent sex-specific patterns [20, 21]. Systemic inflammation is also reported to affect Klotho signalling [22, 23], while in kidney disease α-Klotho may exert vaso- and renoprotective effects [16, 24].

SIRT1 is an NAD + -dependent histone deacetylase that acts as an intracellular energy regulator; through deacetylation of key mediators (e.g., FOXO, NF-κB, p53) it can modulate pathways tied to oxidative stress, inflammation, DNA repair, apoptosis, autophagy and mitochondrial function [25, 26].

Metabolic and inflammatory processes are implicated in development of post-KTx complications, while underlying pathobiological processes are also affected by maintenance immunosuppressive treatment in KTRs [2, 27, 28]. The accelerated aging and chronic inflammation described in the setting of CKD implicate potential roles for both α-Klotho and SIRT1 in disease progression [29, 30]. However, the relationships between α-Klotho, SIRT1, and inflammatory markers in KTRs are not well understood. This study investigated serum concentrations of α-Klotho, SIRT1, and high-sensitivity interleukin-6 (hsIL-6) in KTRs and their associations with hospitalization outcomes and clinical characteristics.

## Materials and methods

### Study design

This was an observational cohort study that enrolled 127 KTRs under routine ambulatory care at the Nephrology and Transplantology Department of the University Hospital in Kraków, Poland, between 1 September 2016 and 30 June 2019. The median (IQR) follow-up duration was 29 [25–32] months. All patients provided written informed consent prior to study participation. This study was conducted in accordance with Declaration of Helsinki and ICH/GCP guidelines and represents an extension of our prior work and institutional research into circulating biomarkers in KTRs. Approval from the local Bioethics Committee was granted in extension of the original biomarker assessment study and updated for patient follow-up (Bioethics Committee Decision No. 1072.6120.202.2022).

### Data collection

Demographic and clinical data were collected at the time of patient enrolment and over follow-up based on manual chart review. Longitudinal assessments were obtained through screening of electronic and paper-based medical records from consecutive ambulatory clinic visits by physicians, at pre-defined 3-to-6-month intervals. The main outcomes assessed over follow-up included: (i) hospitalization at the University Hospital departments for any cause; (ii) all-cause mortality; and (iii) death-censored graft loss, defined as graft dysfunction requiring permanent return to dialysis or re-transplantation listing. Study outcome data was gathered based on direct patient, family or dialysis centre contact, when available. Individual record censoring occurred at the date of death, dialysis transfer or last ambulatory care visit assessment.

### Definitions

Hypertension was defined as outpatient visit blood pressure measurement ≥140/90 mmHg or recorded use of antihypertensive medications. Dyslipidaemia was defined based on existing medical record or use of lipid-lowering agents. Similarly, diabetes was identified based on pre-existing records or use of glucose-lowering therapy and/or biochemical data according to Polish Diabetes Society guidelines. Coronary artery disease (CAD) was defined broadly as a composite outcome of either history of myocardial infarction, stroke/transient ischemic attack, percutaneous coronary intervention or coronary artery bypass grafting, or existing record of chronic coronary syndrome. We did not include heart failure within this definition. Body mass index (BMI) and waist-to-hip ratio (WHR) were calculated based on standard formula. Estimated glomerular filtration rate (eGFR) was calculated using the Chronic Kidney Disease Epidemiology Collaboration (CKD-EPI) 2021 race-free equation via *nephro* package [31]. CKD was classified according to KDIGO guidelines [32].

### Biochemical assessment

We collected peripheral venous blood samples from patients presenting for first study visit following overnight fast. Serum was obtained and stored in serum separator tubes, allowed to clot at room temperature, and then centrifuged. Aliquots were frozen at −70 °C and stored. Assays were conducted later, in batch and under standardized conditions. Procedures were performed by experienced laboratory staff blinded to patient outcomes.

Concentrations of study markers were measured using commercially available enzyme-linked immunosorbent assay (ELISA) kits, in accordance with manufacturer instructions. For α-Klotho (Immuno-Biological Laboratories Co., Ltd., Fujioka, Japan; Catalog No. 27998) sensitivity was 6.15 pg/mL; measurement range 93.75–6,000 pg/mL; intra-assay coefficient of variation (CV) < 3.5% and inter-assay CV was <11.4%. For sirtuin-1 (Wuhan EIAab Science Co., Ltd., Wuhan, China; Catalog No. E3949h) sensitivity <32 pg/mL; detection range 78–5,000 pg/mL; intra-assay CV was <4.3% and inter-assay CV was <7.2%. For high-sensitivity Interleukin-6 (hsIL-6; Quantikine HS ELISA Human IL-6 Immunoassay; R&D Systems, Inc., Minneapolis, MN, USA; Catalog No. HS600B) the mean minimum detectable dose was 0.039 pg/mL; reference range: 0.447–9.96 pg/mL; intra-assay CV was <7.8% and inter-assay CV was <9.6%.

Routine biochemical tests, including serum creatinine, were measured using standardized methods on automated analysers, as per standard laboratory protocol (Hitachi 917, Hitachi, Tokyo, Japan; Modular P, Roche Diagnostics, Mannheim, Germany).

### Statistical analysis

Analyses were performed in R 4.5.3 (R Core Team, 2026; R Foundation for Statistical Computing, Vienna, Austria). Continuous and categorical variables were summarized as median with interquartile range (IQR) and counts with proportion (N, %), respectively. Variable distributions were assessed with visual inspection of density plots and Shapiro-Wilk test. For between-group comparisons we utilized exact Wilcoxon rank-sum tests for continuous variables, whereas Fisher’s test was employed for categorical variable comparison. To assess relationships from a robust perspective, we utilized Spearman’s rank correlation coefficient (rho), with 95% confidence intervals (CI) derived using percentile bootstrap over 2000 replicates.

The relationship between ln (α-Klotho) and final eGFR was modelled using hierarchical linear regression approach. CIs for coefficients were derived using bootstrap methods. We performed influence diagnostics and Cohen’s f^2^ was utilized to quantify effect size. For additional sensitivity, we confirmed linearity of relationships based on alternative restricted cubic spline (3 knot; P = 0.07 for non-linearity).

Due to overdispersion of hospitalization counts, we considered different modelling approaches. Overall, we utilized quasi-Poisson regression, with log-transformed follow-up time as offset variable. To assess whether ln (α-Klotho) was independently associated with outcome after adjustment for all candidate predictors, we fitted a fully-adjusted bias-reduced Poisson model via brglm2 package. Additionally, non-parametric bootstrap and a negative binomial sensitivity model were considered. The Firth model was refitted with baseline CKD-EPI eGFR added as a covariate to further test if serum α-Klotho provides additional information over eGFR. Multicollinearity was assessed with variance inflation factors. Statistical tests were two-tailed and we treated P < 0.05 as statistically significant.

## Results

### Baseline characteristics of the study cohort

**FIGURE 1:**
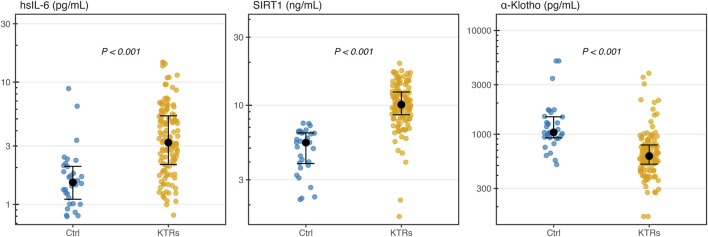
Comparison of serum concentrations of α-Klotho, SIRT1, and hsIL-6 in KTRs and controls. Abbreviations: hsIL-6, high-sensitivity interleukin-6; SIRT1, sirtuin-1; KTRs, kidney transplant recipients; Ctrl, healthy controls. Notes: Each point corresponds to assay value for a specific biomarker candidate in an individual kidney transplant recipient at baseline visit. Median and interquartile range are shown as horizontal black lines with central point. Cross group comparison P values are shown above each panel and are based on Wilcoxon rank-sum tests.

This study included 127 KTRs who were followed for a median (IQR) time of 29 [25–32] months. Most patients were of male gender (N = 86, 67.7%) and middle-aged, with median (IQR) age of 54 [33–52] years. We compared serum α-Klotho concentrations between KTRs and 32 healthy controls (16 male [50.0%]; mean age 50 years, age range 29–74 years). We observed lower circulating α-Klotho levels in serum of KTRs, with median (IQR) values of 616 (514–788) pg/mL versus 1,042 (922–1,472) pg/mL (P < 0.001), which correspond to an estimated difference of 463 (95% CI 346–605) pg/mL (see Figure 1).

For descriptive comparison of clinical characteristics, we stratified patients using a 75th-percentile cut-off (788 pg/mL) into “low” (n = 95) and “high” (n = 32) α-Klotho groups (see Table 1). However, for inferential analyses, serum α-Klotho was modelled as a continuous variable with natural log transformation due to distributional considerations. Both “low” and “high” α-Klotho groups were comparable in age, sex, body mass indices, metabolic parameters, smoking status, and baseline eGFR. Interestingly, hypertension was more frequently recorded in the low α-Klotho group (P = 0.016). The high α-Klotho group also showed lower log-transformed hsIL-6 levels, with median (IQR) values of 0.81 (0.37–1.20) versus 1.31 (0.94–1.73) (P < 0.001). Respectively, higher SIRT1 levels, with median (IQR) values of 11.46 (9.78–14.48) versus 9.82 (8.11–11.52) ng/mL were recorded in the “high” α-Klotho groyp, as compared to “low” α-Klotho group (P = 0.010). Of note, this difference in SIRT1 concentrations was not robust to the cut-off, as detailed in Section *Relationships between clinical characteristics and α-Klotho and SIRT1 concentrations in serum of KTRs*.

**TABLE 1:** Demographic and clinical characteristics of kidney transplant recipients stratified by serum α-Klotho levels.

Characteristic	High α-Klotho (n = 32)	Low α-Klotho (n = 95)	P Value
Age, years	53.0 (40.0–59.3)	56.0 (42.0–61.5)	0.394
Sex, male, N (%)	23 (71.9)	63 (66.3)	0.664
BMI (kg/m^2^)	24.27 (23.55–27.22)	26.30 (23.05–29.51)	0.176
Waist-to-hip ratio	0.93 (0.87–0.99)	0.94 (0.86–1.00)	0.780
Mean arterial pressure, mmHg	103.17 (91.83–110.17)	98.67 (93.00–108.00)	0.311
Smoking status, N (%)	​	​	0.360
Never	25 (78.1)	71 (76.3)	​
Former	3 (9.4)	16 (17.2)	​
Current	4 (12.5)	6 (6.5)	​
Diabetes mellitus, N (%)	11 (34.4)	23 (24.2)	0.356
Hypertension, N (%)	26 (81.2)	91 (95.8)	0.016
Dyslipidaemia, N (%)	5 (15.6)	13 (13.7)	0.774
Coronary artery disease, N (%)	4 (12.5)	21 (22.1)	0.309
HbA1c, %	5.90 (5.40–6.05)	5.75 (5.47–6.30)	0.778
Fasting glucose, mmol/L	5.60 (5.05–6.00)	5.38 (4.94–5.82)	0.286
Serum creatinine, baseline, µmol/L	108.0 (89.8–141.0)	130.0 (95.0–169.5)	0.070
Follow-up, months	29.43 (27.31–31.75)	28.50 (24.42–31.83)	0.388
Metabolic syndrome, N (%)	9 (32.1)	21 (26.2)	0.626
SIRT1, ng/mL	11.46 (9.78–14.48)	9.82 (8.11–11.52)	0.010
ln (hsIL-6)	0.81 (0.37–1.20)	1.31 (0.94–1.73)	<0.001
ln (α-Klotho)	6.89 (6.78–7.34)	6.34 (6.10–6.47)	<0.001

Abbreviations: BMI, body mass index; HbA1c, glycated hemoglobin; hsIL-6, high-sensitivity interleukin-6; SIRT1, sirtuin-1.

Data are shown as median (interquartile range) unless otherwise stated. Stratification follows a 75th-percentile cut-off at 788 pg/mL. This cut-off is descriptive only; all inferential analyses utilize serum α-Klotho levels as a continuous variable.

Between-group comparison is based on exact Wilcoxon rank-sum tests and Fisher’s exact test for continuous and categorical variables, respectively. For body-mass indices and mean arterial hypertension data were available for n = 123, for HbA1c% n = 71 patients and for fasting glucose n = 110, respectively.

### Relationships between clinical characteristics and α-Klotho and SIRT1 concentrations in serum of KTRs

For monotonic relationship analyses, we followed Cohen’s approach to interpretation of Spearman’s correlation strength (rho = 0.10, weak; rho = 0.30, moderate; rho = 0.50, strong). We observed that serum α-Klotho was consistently associated with allograft function across all timepoints: baseline eGFR (rho = 0.304), follow-up T1 (rho = 0.315), and follow-up T2 (rho = 0.29). Circulating α-Klotho in serum was inversely correlated with hsIL-6 levels (rho = −0.39, 95% CI -0.60–0.15, P = 0.002). Importantly, this association was also consistent in alternative analysis with α-Klotho categorization approaches (P = 0.001 for the 75th-percentile cut-off, P = 0.026 for the median cut-off).

By contrast, the α-Klotho-SIRT1 relationship was weaker and cut-off dependent. In continuous analysis, Spearman rho was estimated at 0.21 (95% CI -0.04–0.44, P = 0.098). Group differences are reported descriptively in Table 1 (P = 0.010 for the 75th-percentile cut-off), though were not preserved using a median split (P = 0.31). The SIRT1 finding should be interpreted with caution and requires validation. Other monotonic relationships between α-Klotho and clinical features were weak: HbA1c (rho = −0.140), BMI (rho = −0.087), mean arterial pressure (rho = 0.099), waist-to-hip ratio (rho = 0.020), and age (rho = 0.006).

### Relationship between changes in kidney allograft function over follow-up

At baseline, median (IQR) eGFR was 57.4 (41.7–72.9) mL/min/1.73 m^2^, with a median change of 0 (−10.4–7.1) mL/min/1.73 m^2^ over follow-up. Most KTRs (N = 103, 80%) were characterized with stable graft function, which we defined as absolute change within 15 mL/min/1.73 m^2^. At the last available follow-up visit, KDIGO stage distribution was as follows: G1 (11.8%), G2 (35.4%), G3a (20.5%), G3b (12.6%), G4 (11.8%), and G5 (7.9%).

**FIGURE 2:**
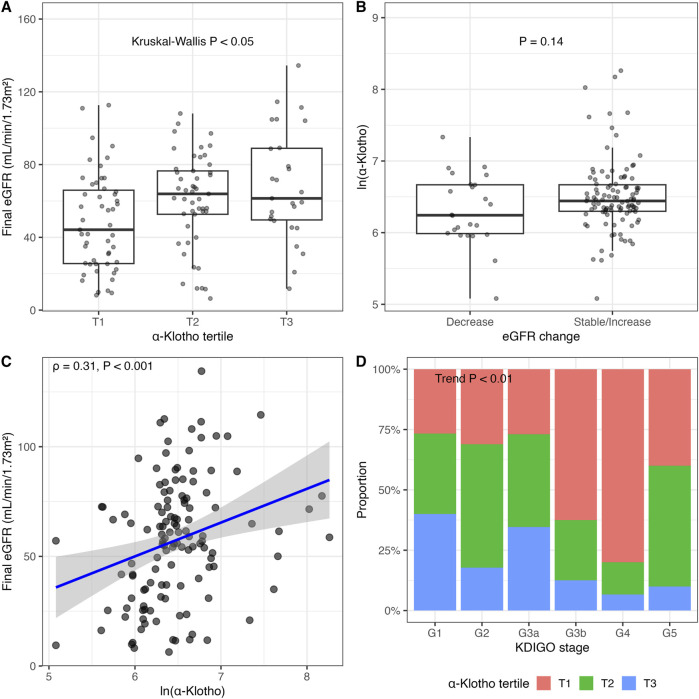
Relationship between serum α-Klotho levels and kidney function in kidney transplant recipients. Abbreviations: eGFR, estimated glomerular filtration rate; KDIGO, Kidney Disease: Improving Global Outcomes; ln, natural logarithm; T1, tertile 1 (lowest α-Klotho); T2, tertile 2; T3, tertile 3 (highest α-Klotho). Notes: **(A)** illustrates distribution of final eGFR across serum α-Klotho tertiles based on box plot, with P values based on Kruskal-Wallis. **(B)** compares log-transformed α-Klotho concentrations according to eGFR change based on Wilcoxon exact test. **(C)** illustrates the monotonic relationship with Spearman correlation coefficient (rho), while shaded area corresponds to standard error uncertainty. **(D)** shows distribution of α-Klotho tertiles across KDIGO stages, with P value based on permutation-based trend test (using 10,000 resamples).

We observed that when patients were stratified by serum concentrations of α-Klotho categorized into tertiles, they differed by eGFR (P = 0.010; Figure 2A). Specifically, patients in the lowest tertile had a median (IQR) final eGFR of 44.2 (25.6–65.9) mL/min/1.73 m^2^, which is markedly lower than the second tertile (63.9 (52.7–76.5); P = 0.048) and the third tertile (61.4 (49.6–89.0); P = 0.024). Patients with eGFR decline also showed numerically lower median ln (α-Klotho) (P = 0.14; Figure 2B). Further, the continuous monotonic relationship between ln (α-Klotho) and final eGFR was formally significant (rho = 0.28, P = 0.002; Figure 2C), and the distribution of α-Klotho tertiles across KDIGO stages also supported a significant trend association (P = 0.003; Figure 2D).

We then constructed iterative linear regression models with progressive covariate adjustment (see Table 2). In the unadjusted model, each unit increase in ln (α-Klotho) was associated with 15.4 mL/min/1.73 m^2^ higher final eGFR (95% CI 6.7–25.4). The association was preserved after demographic adjustment (Beta 15.3, 95% CI 7.3–25.8) and after additional adjustment for BMI and diabetes status (Beta 14.8, 95% CI 6.2–25.6). However, the effect size was modest (f^2^ = 0.083). In additional sensitivity analysis after excluding high-influence observations, the relationship was consistently observed (Beta 15.4, 95% CI 3.1–27.7, P = 0.015). We also confirmed the plausible linearity of the ln (α-Klotho) effect using comparison with a three-knot restricted cubic spline model (P = 0.07 for non-linearity).

**TABLE 2:** Relationship between circulating α-Klotho and last available eGFR over follow-up.

Model	Beta (95% CI)	Adj. R^2^
Baseline (unadjusted)	15.41 (6.73–25.44)	0.070
Baseline + age + sex	15.31 (7.28–25.81)	0.057
Baseline + BMI + diabetes	14.80 (6.22–25.59)	0.038

Abbreviations: Adj., adjusted; BMI, body mass index; CI, confidence interval; eGFR, estimated glomerular filtration rate.

Beta coefficients should be interpreted as change in final eGFR, per 1 unit change in α-Klotho on natural log scale. 95% Cis are based on bootstrap.

### Relationship between α-Klotho, SIRT1 and all-cause hospitalization

Over 274 person-years of follow-up, we recorded 153 inpatient admissions that occurred among 54 KTRs (42.5%); 73 (57.5%) were not hospitalized during follow-up. Of note, we recorded hospitalizations that occurred within the setting of the University Hospital. Recurrent admissions ranged from 1 to 12 per patient, with a median (IQR) of 1 [1, 2] admissions. In age-adjusted quasi-Poisson regression (see Table 3), higher ln (α-Klotho) was significantly associated with lower hospitalization rate (IRR 0.38, 95% CI 0.19–0.74, P = 0.006). Coronary artery disease was also associated with higher rate (IRR 2.51, 95% CI 0.98–6.14, P = 0.049), while sex, diabetes, hypertension, dyslipidaemia, mean arterial pressure, BMI, ln (hsIL-6), and SIRT1 were not significant predictors.

**TABLE 3:** Predictors of all-cause hospitalization in kidney transplant recipients.

Predictor	Crude IRR (95% CI)	P	Age-adj. IRR (95% CI)	Age-adj. P
Sex, male	0.71 (0.34–1.52)	0.366	0.71 (0.34–1.52)	0.361
Diabetes mellitus	1.03 (0.42–2.27)	0.937	1.05 (0.42–2.31)	0.916
Hypertension	0.93 (0.30–4.62)	0.912	0.93 (0.30–4.69)	0.919
Dyslipidaemia	1.08 (0.33–2.74)	0.887	1.08 (0.32–2.76)	0.884
Coronary artery disease	1.91 (0.84–3.94)	0.099	2.51 (0.98–6.14)	0.049
BMI, per kg/m^2^	1.02 (0.94–1.10)	0.541	1.03 (0.95–1.10)	0.509
MAP, per mmHg	1.00 (0.97–1.03)	0.783	1.00 (0.98–1.03)	0.763
ln (hsIL-6)	1.39 (0.81–2.37)	0.230	1.41 (0.82–2.39)	0.205
SIRT1, per ng/mL	0.91 (0.82–1.02)	0.117	0.91 (0.82–1.02)	0.117
ln (α-Klotho)	0.37 (0.19–0.74)	0.005	0.38 (0.19–0.74)	0.006

Abbreviations: Adj., adjusted; BMI, body mass index; CI, confidence interval; hsIL-6, high-sensitivity interleukin-6; IRR, incidence rate ratio; MAP, mean arterial pressure; SIRT1, sirtuin-1.

Predictors are based on a quasi-Poisson regression model with offset based on log (follow-up). Additionally, we performed a fully-adjusted Firth-corrected sensitivity analysis that is available in the Supplementary Material (Supplementary Table S1).

**FIGURE 3:**
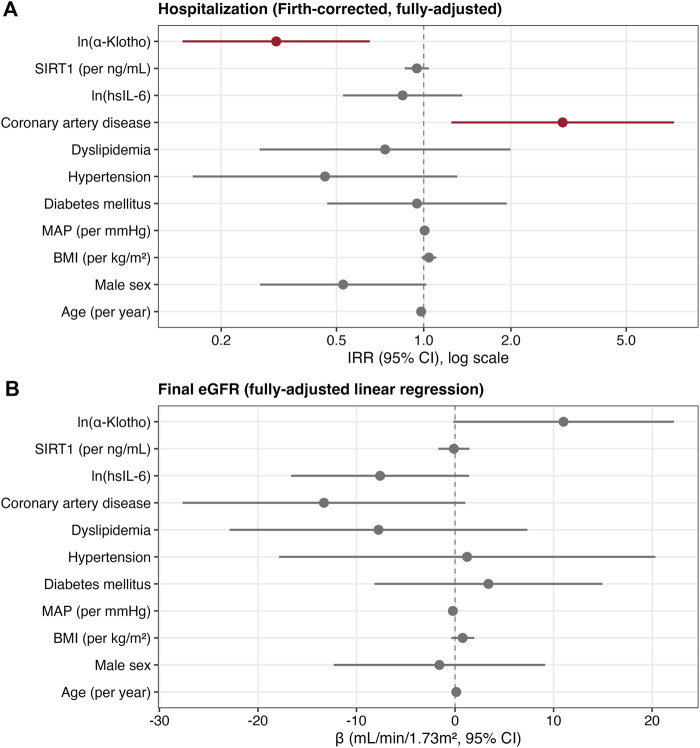
Forest plot of hospitalization risk and final eGFR predictors based on the full multivariable model. Abbreviations: BMI, body mass index; CAD, coronary artery disease; CI, confidence interval; eGFR, estimated glomerular filtration rate; IRR, incidence rate ratio; MAP, mean arterial pressure; SIRT1, sirtuin-1. Notes: **(A)** illustrates incidence rate ratios for hospitalization from a fully-adjusted Firth-corrected bias-reduced Poisson model (n = 123, 151 events). **(B)** illustrates linear regression coefficients for final eGFR based on a fully-adjusted linear model. Points and horizontal bars correspond to point estimates with 95% confidence intervals. Coloured points are indicative of predictors with P < 0.05, while grey points indicate non-significant covariates.

To evaluate whether ln (α-Klotho) was independently associated with readmission after adjustment for all candidate predictors, we fitted a fully-adjusted Firth-corrected Poisson model containing age, sex, BMI, mean arterial pressure, diabetes mellitus, hypertension, dyslipidaemia, coronary artery disease, ln (hsIL-6), SIRT1, and ln (α-Klotho), with robust standard errors and log (follow-up time) as offset (Supplementary Table S1; Figure 3A). Although exploratory, ln (α-Klotho) was observed with a maintained, strong inverse relationship with hospitalization rate (IRR 0.31, 95% CI 0.15–0.65, P = 0.002). Similar observations were noted for coronary artery disease (IRR 3.02, 95% CI 1.25–7.30, P = 0.014). These findings were consistent across different, alternative model constructs, both using a non-parametric bootstrap of the same model (R = 1,000) with a 95% CI of 0.10–0.66, as well as a negative binomial regression model (similarly, the estimated IRR was 0.32 95% CI 0.14–0.73, P = 0.005). In model diagnostics, all variance inflation factors were <2, which suggesting limited confounding by collinearity.

To further investigate whether α-Klotho may provide additional information over eGFR monitoring, we refitted the fully adjusted Firth model with baseline eGFR added as a covariate. Consistently, ln (α-Klotho) consistently demonstrated an independent inverse association with hospitalization rate (IRR 0.37, 95% CI 0.20–0.69, P = 0.002), even after adjustment for baseline eGFR. At the same time, baseline eGFR was a strong predictor of hospitalization (IRR 0.97 per mL/min/1.73 m^2^; P < 0.001).

## Discussion

The salient findings of this observational study are that (i) serum α-Klotho concentrations are significantly reduced in KTRs and (ii) are independently associated with future graft function, (iii) inversely correlated with circulating inflammatory burden, and (iv) inversely associated with all-cause hospitalization, even after adjustment for baseline kidney function. Our results extend evidence from prior work and recent meta-analyses that have reported reduced serum α-Klotho levels after kidney donation, while an increase of α-Klotho was observed in individuals post-KTx [53]. Our findings place α-Klotho as a candidate supplemental marker that may add information to standard eGFR-based risk stratification in the post-KTx setting.

In this exploratory study we observed that higher serum α-Klotho concentrations were associated with preserved graft function over follow-up, an observation consistent across multivariable specifications for last available eGFR. This aligns with reports from other KTR cohorts, in which higher circulating α-Klotho was tied to improvement in renal function over time [54–57].

Importantly, the lower hospitalization rate associated with higher α-Klotho remains a novel and clinically relevant finding, strengthened by consistent risk attribution in different models, even when baseline eGFR was added as a covariate to the fully adjusted model. Preliminary reports suggest α-Klotho deficiency may be detectable at very early stages of CKD [58, 59], though few studies have investigated circulating α-Klotho levels in KTRs, with mixed results [60]. Other research groups have reported associations between α-Klotho levels and glomerular function [56, 57], though variability of α-Klotho concentrations at different post-transplant timepoints has been observed, with decremental trends over time [60, 61]. While baseline eGFR and age remain important confounders and determinants of graft function [33], our analyses suggest serum α-Klotho is an independent predictor of future eGFR and may provide supplemental risk stratification value.

While Klotho functions as a co-receptor for FGF-23 signalling, its full biological roles and protective mechanisms remain unelucidated [13, 34]. High Klotho expression has been demonstrated in the kidney [35], with this organ identified as a major source of circulating α-Klotho [36]. In experimental models of transgenic mice, Klotho overexpression was tied to improved renal function, reduced calcification and higher phosphaturia [14, 17, 24]. In patients with autosomal dominant polycystic kidney disease, serum α-Klotho was associated with cyst size and renal growth [37]. On a cellular level, Klotho is likely to exert anti-apoptotic [38] and anti-fibrotic [39] effects.

We observed higher prevalence of hypertension in patients with lower α-Klotho levels, alongside an inverse correlation with circulating hsIL-6 levels that was robust to both alternative dichotomizations and continuous analysis. On a theoretical level, cytokines such as TNF-α and IL-6 may suppress α-Klotho expression in the kidney [40–42], and evidence from murine studies suggests Klotho acts as a negative regulator of NF-κB-related inflammation [43]. Oxidative stress, cellular damage and fibrosis due to events such as ischemia-reperfusion are likely to occur post-KTx, with experimental data supportive of a protective role of Klotho [44]. In peritoneal dialysis patients, lower circulating Klotho levels have been associated with higher markers of oxidative stress and inflammation (8-isoprostane and IL-6), although this relationship was not retained in multivariable models [45]. Previous studies in haemodialysis patients have shown that lower Klotho is associated with CV events, in a manner independent of common risk factors and other covariates [46]. Since Klotho levels are closely tied to age and renal function, understanding inter-relationships between these factors appears crucial for clinical interpretation. Taken together, α-Klotho deficiency is likely to be an important player in the development of kidney disease and may also promote vascular calcification [34, 47, 48, 58].

**FIGURE 4:**
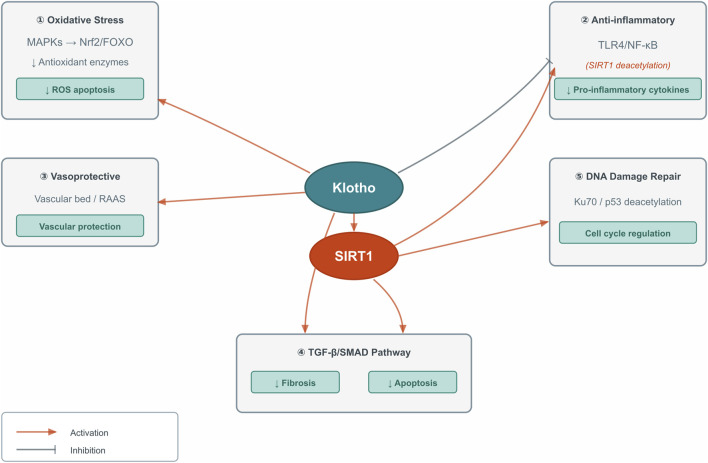
Proposed mechanistic pathways linking Klotho and SIRT1 with cellular protection. Notes: The figure illustrates potential interplay between mechanistic pathways by which Klotho and SIRT1 could exert cytoprotective effects [62–64]. Orange arrows correspond to activating signals, while blunted lines indicate inhibitory activity. (1) Oxidative stress mechanism: Klotho activates MAPKs, promotes Nrf2/FOXO, which leads to antioxidant enzyme expression and reduction in ROS induced apoptosis [62, 65]. (2) Anti-inflammatory mechanism: Klotho inhibits TLR4/NF-κB signaling, reducing pro-inflammatory cytokine production, potentially synergistic with SIRT1-mediated deacetylation of NF-κB [66, 67]. (3) Vasoprotective mechanism: Klotho may act as a hormone and upstream regulator of SIRT1 in the vascular bed, with interaction with renin-angiotensin-aldosterone system [50, 63]. (4) TGF-β/SMAD pathway: Klotho and SIRT1 modulate signaling to attenuate fibrotic and apoptotic responses [52, 64]. (5) DNA damage repair pathways: SIRT1 deacetylates Ku70 and modulates p53 to regulate cell cycle [68, 69].

Theoretical justification for anti-inflammatory (e.g., via inhibition of NF-κB signalling and M2 polarization [49]) effects of SIRT1 is increasing. A schematic representation of the potential, cytoprotective pathways involving α-Klotho and SIRT1 is shown in Figure 4 [14, 50]. Acute rejection studies have also reported SIRT1 decline [51]. However, the descriptive group difference in serum SIRT1 between high and low α-Klotho strata observed at present was not retained in models with continuous predictor form, as well as in categorization approaches. Our continuous analysis did not provide sufficient evidence to speculate regarding an α-Klotho-SIRT1 relationship. This null finding may reflect threshold effects, statistical noise, or a combination of both.

The systemic bioactivity of Klotho may be dependent on both membrane-bound and soluble isoform activity. Vasoprotective effects of soluble α-Klotho have been reported, alongside its capacity to inhibit growth factor pathways driving towards pro-fibrotic transition [24, 52]. However, cross-population differences in serum α-Klotho concentrations may affect inter-assay variance and comparability [36, 70]. While our findings align with some adult and paediatric studies [71, 72], research in dialysis and CKD populations shows inconsistent results [73, 74]. These discrepancies may reflect methodological differences, sample heterogeneity, or the complex inflammatory environment in kidney disorders.

The limitations of our study need to be emphasized. First, we measured the level of circulating candidate markers in serum at a single post-KTx timepoint. Caution in interpretation is required due to the declining levels of α-Klotho over time post-KTx, as well as methodological considerations that relate to freeze-thaw cycle and assay standardization [60, 70]). Moreover, the observational design precludes any causal inference at present. At the same time, reverse causation remains a relevant concern, in that impaired graft function may attenuate α-Klotho levels; therefore, greater mechanistic understanding of potential interactions from experimental models is crucial to build the theoretical framework for Klotho interpretation. Third, it should be stated that serum α-Klotho shows substantial inter-individual variation, while no standardized ELISA is widely accepted. We cannot, therefore, recommend the use of a pragmatic clinical cut-off, although research is encouraged and the use of percentile-based cut-offs seems preferable. Additionally, there are multiple potential confounding factors and exposures, such as diet [75], alcohol consumption [76], exercise [77] or stress [78]. Lastly, the modest sample size, event rate and exploratory nature of this study merit additional caution when interpreting our results on a causal level.

To summarize, in this observational cohort of KTRs, we observed that higher circulating α-Klotho levels were independently associated with reduced all-cause hospitalization rates, even after adjustment for baseline eGFR, and were inversely tied to hsIL-6. Higher KDIGO stage was also associated with lower serum α-Klotho concentrations. Our findings extend the current work in the field that supports circulating α-Klotho as a candidate supplemental marker for the KTx setting. Our findings also indirectly support the ongoing interest into mechanistic aspects of therapies that are hypothesized to modulate α-Klotho expression, which includes renin-angiotensin-aldosterone system inhibitors [79], active vitamin D analogues such as calcitriol and paricalcitol [80], and mTOR inhibitors such as sirolimus and everolimus [81, 82]. Direct strategies, such as recombinant soluble α-Klotho administration, remain highly experimental. Importantly, mechanistic speculation on clinical utility requires high caution. Future prospective, large studies with standardized assays and serial α-Klotho measurements remain of importance to replicate and validate the associations investigated at present. At the same time, experimental studies that will clarify mechanistic relationship underlying α-Klotho relationship with inflammation, renal disease and metabolic dysfunction are warranted.

## Data Availability

The raw data supporting the conclusions of this article will be made available by the authors, without undue reservation.
