# Supplementary material for: Serum α-Klotho and SIRT1 - Relationship with graft function, inflammation and hospitalization rates in kidney transplant recipients
Source: Transpl Int. 2026 Jun 2;39:16186. doi: 10.3389/ti.2026.16186 (PMC13269048; doi:10.3389/ti.2026.16186)
Supplement: Supplementary file 1 [file Table1.docx]

Capsule sentence summary.

**In 127 kidney transplant recipients, lower serum α-Klotho was tied to worse graft function and a higher rate of all-cause hospitalization, independently of baseline eGFR, supporting its role as a candidate supplemental biomarker.**

Table S1.Firth-corrected fully-adjusted Poisson regression for hospitalization.

| **Predictor** | **IRR (95% CI)** | ***P* value** |
| --- | --- | --- |
| Age, year | 0.98 (0.96-1.01) | 0.119 |
| Sex, Male | 0.53 (0.27-1.02) | 0.058 |
| BMI, kg/m² | 1.04 (0.98-1.11) | 0.172 |
| MAP, mmHg | 1.01 (0.98-1.03) | 0.592 |
| Diabetes mellitus, Yes | 0.95 (0.46-1.93) | 0.883 |
| Hypertension, Yes | 0.46 (0.16-1.31) | 0.144 |
| Dyslipidaemia, Yes | 0.74 (0.27-1.99) | 0.546 |
| Coronary artery disease, Yes | 3.02 (1.25-7.30) | 0.014 |
| ln(hsIL-6) | 0.85 (0.53-1.36) | 0.489 |
| SIRT1, ng/mL | 0.95 (0.86-1.04) | 0.264 |
| ln(α-Klotho) | 0.31 (0.15-0.65) | 0.002 |

This summary is based on a bias-reduced Poisson regression model using brglm2 package with HC0 robust standard errors. Model utilizes log-transformed follow-up time as offset. Data using N=123 patients with complete covariate data, with 151 events.
